# Correction: Frequency of relapse for severe acute malnutrition and associated factors among under five children admitted to health facilities in Hadiya Zone, South Ethiopia

**DOI:** 10.1371/journal.pone.0304733

**Published:** 2024-05-29

**Authors:** Abera Lambebo, Dessalegn Tamiru, Tefera Belachew

The second author’s name is spelled incorrectly. The correct name is: Dessalegn Tamiru. The correct citation is: Lambebo A, Tamiru D, Belachew T (2021) Frequency of relapse for severe acute malnutrition and associated factors among under five children admitted to health facilities in Hadiya Zone, South Ethiopia. PLoS ONE 16(3): e0249232. https://doi.org/10.1371/journal.pone.0249232.
