# Supplementary material for: Frequency of relapse for severe acute malnutrition and associated factors among under five children admitted to health facilities in Hadiya Zone, South Ethiopia
Source: PLoS One. 2021 Mar 25;16(3):e0249232. doi: 10.1371/journal.pone.0249232 (PMC7993841; doi:10.1371/journal.pone.0249232)
Supplement: Attachment — Submitted filename: rebuttal letter .docx [file pone.0249232.s002.docx]

| Reviewer or editor | Parts of document | Comment given | Response by authors |
| --- | --- | --- | --- |
| Editors | Methodological area | Please include the name of 20 health post | Accepted and corrected |
| Editors | Methodological area | Please include additional information regarding the survey or questionnaire | Accepted and corrected |
| Editors | Ethical clearance | Please also add this information to your ethics statement in the online submission form. | Accepted and corrected |
| Editors | About data | upload your study’s minimal underlying data | uploaded |
| Editors | About title | Please amend either the title on the online submission | amended |
| Editors | About abstract | Please amend either the abstract on the online submission | amended |
| Reviewer #1 | On discussion | add some risk factors of malnutrition: es HIV, low socio-economic level, malaria etc | Not amended there is no information on registration book for SAM. |
| Reviewer #2 | Methodology | definitions for SAM and relapse, briefly illustrate the used treatment protocol, clarify discharge criteria (% of weight gain?). | Amended and corrected |
| Reviewer #3: | On discussion | in the discussion section such as:  Relapse after severe acute malnutrition: A systematic literature review and secondary data analysis | As this is per revied some were addressed and systematic is not included in discussion |
