# Supplementary material for: Frequency of relapse for severe acute malnutrition and associated factors among under five children admitted to health facilities in Hadiya Zone, South Ethiopia
Source: PLoS One. 2021 Mar 25;16(3):e0249232. doi: 10.1371/journal.pone.0249232 (PMC7993841; doi:10.1371/journal.pone.0249232)
Supplement: Attachment — Submitted filename: rebuttal letter -3.docx [file pone.0249232.s003.docx]

**A rebuttal letter that responds to each point raised by the academic editor and reviewer**

| Reviewer or editor | Parts of document | Comment given | Response by authors |
| --- | --- | --- | --- |
| Editors | Reference | Please review your reference list to ensure that it is complete and correct | Accepted and corrected |
| Reviewer #1 | General | Authors wrote an interesting paper and I suggest to accept the paper | - |
| Reviewer #2 | - Introduction - In operational definitions - In strength and limitation part | - In the last paragraph of introduction - specify what is the “clinical sign”? bilateral edema? - Several important factors were not included | Amended and corrected |
